# Fine Mapping to Identify the Functional Genetic Locus for Red Coloration in *Pyropia yezoensis* Thallus

**DOI:** 10.3389/fpls.2020.00867

**Published:** 2020-06-23

**Authors:** Xinzi Yu, Lu Wang, Kuipeng Xu, Fanna Kong, Dongmei Wang, Xianghai Tang, Bin Sun, Yunxiang Mao

**Affiliations:** ^1^Key Laboratory of Marine Genetics and Breeding (Ministry of Education), Ocean University of China, Qingdao, China; ^2^College of Marine Life Sciences, Ocean University of China, Qingdao, China; ^3^Key Laboratory of Utilization and Conservation of Tropical Marine Bioresource (Hainan Tropical Ocean University), Ministry of Education, Sanya, China

**Keywords:** *Pyropia yezoensis*, mutant, red coloration, QTL-seq, KASP, RNA-seq, fine mapping

## Abstract

*Pyropia yezoensis*, commonly known as “Nori” or “Laver” is an economically important marine crop. In natural or selected populations of *P. yezoensis*, coloration mutants are frequently observed. Various coloration mutants are excellent materials for genetic research and study photosynthesis. However, the candidate gene controlling the *Pyropia* coloration phenotype remains unclear to date. QTL-seq, in combination with kompetitive allele-specific PCR (KASP) and RNA-seq, can be generally applied to population genomics studies to rapidly identify genes that are responsible for phenotypes showing extremely opposite traits. Through cross experiments between the wild line RZ and red-mutant HT, offsprings with 1–4 sectors chimeric blade were generated. Statistical analyses revealed that the red thallus coloration phenotype is conferred by a single nuclear allele. Two-pair populations, consisting of 24 and 56 wild-type/red-type single-genotype sectors from F1 progeny, were used in QTL-seq to detect a genomic region in *P. yezoensis* harboring the red coloration locus. Based on a high-quality genome, we first identified the candidate region within a 3.30-Mb region at the end of chromosome 1. Linkage map-based QTL analysis was used to confirm the candidate region identified by QTL-seq. Then, four KASP markers developed in this region were used to narrow down the candidate region to a 1.42-Mb region. Finally, we conducted RNA-seq to focus on 13 differentially expressed genes and further predicted *rcl-1*, which contains one non-synonymous SNP [A/C] in the coding region that could be regulating red thallus coloration in *P. yezoensis*. Our results provide novel insights into the underlying mechanism controlling blade coloration, which is a desirable trait in algae.

## Introduction

Blade color mutations endow plants with various colors that can influence physiological changes in plants and increase the economic value of agricultural products and ornamental plants (Yamasaki, 1997; Xing et al., 2014). Blade color is also an effective marker to identify the hybridization in genetic breeding (Luo et al., 1991; Zhang et al., 2016). Thus blade color mutants have sparked the interest of numerous scientists.

So far, with increasing blade color mutants identified such as *Arabidopsis thaliana* (Hsieh et al., 2017) rice (Ma et al., 2017), and Chinese cabbage (Feng et al., 2010; Wang et al., 2014) more genes related to color changes have been identified. The mutation mechanisms of color changes are diverse, and these related genes are associated with chloroplast development and pigment metabolic pathways. Some genes are directly related to pigment biosynthesis/degradation pathways such as chlorophyll (*Chl*) and carotenoids (Fang et al., 2008; Wang P. et al., 2010) other genes indirectly influence the metabolism of pigments through multiple biological pathways (Li et al., 2015; Lv et al., 2015). For example, in rice, *Os07g0558500* is predicted to be the ortholog of *THF1* and directly indispensable for *Chl* degradation (Yamatani et al., 2013). In *A. thaliana, AtPALE1* that encodes a TMP phosphatase has been identified and results in defective chloroplast development that indirectly leads to pale green mutant (Hsieh et al., 2017). *FdC2* encodes a ferredoxin-like protein that mediates electron transfer and indirectly results in yellow-green leaf mutants (Li et al., 2015).

*Pyropia yezoensis*, commonly known as “Nori” or “Laver” and growing in the intertidal zone, is one of the most economically important marine crops in the world and has developed complex regulatory mechanisms for adapting to the environment (Gao et al., 2013). Various spontaneous or artificial coloration mutants of *P. yezoensis* were isolated from cultivated populations such as red mutants, green mutants, and yellow mutants (Yan et al., 2000). These pigment mutants with different colors have various characters that influence photosynthesis, growth rates, and even quality, thus enriching germplasm resources for heredity and breeding of laver (Zhang et al., 2014; Ueno et al., 2017). Since coloration mutants appeared, pigment mutations have been used as markers to simplify the process of breeding and cross selection of improved varieties (Niwa et al., 2003; Niwa et al., 2009). Using hybrid materials from wild-type and coloration mutants, it has been found that conchospores form color-chimeric order tetrads during the first two cell divisions (Ohme and Miura, 1988; Hawley, 2007), which indicated that the members of *Pyropia* undergo meiosis during the first two divisions of the germinating conchospores (Zhou et al., 2007). Color-chimeric order tetrads then further develop into a lot of sectored gametophytic blades (chimeras) consisting of 2–4 sectors. By counting the color sectors, the inheritance patterns of the red coloration loci are elucidated, and it was revealed that the color mutation may be caused by nuclear gene mutation, cytoplasmic mutation, or nuclear cytoplasmic double mutation (Mitman and van der Meer, 1994; Niwa, 2010). Coloration mutants in accordance with Mendel’s laws can be used to elucidate gene interactions and localize economic traits in *Pyropia* (Yan and Aruga, 2000). Germinating conchospores’ ordered tetrad and haploid blade character allowed the identification of gene-centromere distances and linkages between mutant genes (Niwa, 2010). Huang and Yan constructed a genetic linkage map based on 92 SRAP markers using the doubled haploid (DH) population generated by the hybrid of red-type mutant blades and WT blades and identified quantitative trait loci (QTLs) of six economic traits of gametophytic blades of *P. yezoensis* (Huang and Yan, 2019a). These locating researches just located traits at the interval level, but not at the gene level. The alleles underlying *Pyropia* coloration have yet to be identified, thereby limiting the application of coloration mutants in breeding and genetic analysis.

Traditional linkage map-based QTL analysis usually employs a limited number of RFLP, AFLP, SSR, RAPD, or SRAP markers for trait location (Kong et al., 2009). Recently, various technologies by whole-genome sequencing using next-generation sequencing (NGS) provide a high-throughput means to generate large number of makers such as SNPs and InDels. These technologies were applied to more effectively achieve candidate genes identification and location with the development of sequencing such as QTL-seq. QTL-seq is a faster and effective strategy based on SNPs/InDels, which employs NGS technology for whole-genome resequencing of two progeny DNA bulks composed of 20–50 individuals showing extreme opposite trait values in a segregating mapping population to achieve rapid identification of qualitative traits and quantitative traits with major genes (Takagi et al., 2013). The wider applicability and outstanding advantages of QTL-seq over traditional linkage map-based QTL mapping strategies available in diverse plant species have thus been truly achieved (Kayam et al., 2017; Clevenger et al., 2018). Although QTL-seq is convenient and efficiently narrows down the range of candidate QTL regions, it also has many limitations, such as for regions with insufficient meiotic recombination events, the causal genes may not be identified (Wen et al., 2019). To overcome these limitations, this study used QTL-seq, developing kompetitive allele-specific PCR (KASP) technology, and RNA-seq to detect red thallus coloration in *P. yezoensis*. Currently, genetic studies in macroalgae have not been conducted as fast as higher plants due to the lack of high-quality genome information, appropriate genetic materials, and research methods applied in macroalgae, so the application of this strategy for the exploration of functional genes in algae has not been reported.

To fine map the alleles underlying blade coloration, a combined method consisting of QTL-seq, KASP, and RNA-seq based on crossing was performed. The results of fine mapping could serve as a valuable resource for elucidating the molecular mechanisms of blade coloration and provide useful markers for the genetic analysis of Bangiales.

## Materials and Methods

### Plant Materials

**FIGURE 1:**
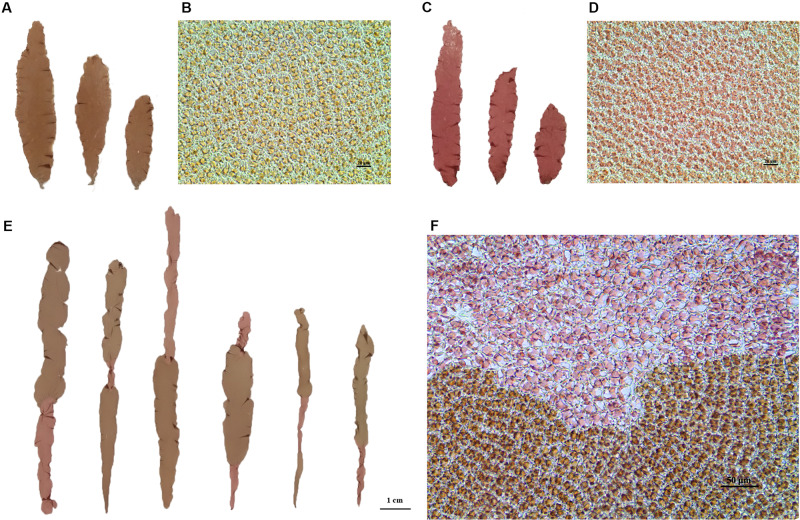
**(A,C)** Blades of wild maternal RZ (left) and mutant paternal HT (right) as of *P. yezoensis*. **(B,D)** Microscopic cells were observed under a 40 × objective microscope. Left, RZ; right, HT. **(E)** Examples of sectored blades from the F1 generation. **(F)** Microscopic observation of the boundary between two color sectors.

Two lab-cultured genetically pure lines of *P. yezoensis*, the WT PYL201306-440 (RZ) (Figures 1A,B) and the red-mutant PYL201306-438 (also named as HT) (Figures 1C,D), were used in the present study. The spontaneous red-mutant HT was isolated from the WT PYL-349 (4–7). The gametophytes were cultured in bubbling natural seawater with Provasoli’s enrichment solution medium (PES) under 50 μmol photons m^–2^⋅s^–1^ at 8 ± 1°C and a 12:12 light:dark (L:D) photoperiod. Free-living conchocelis filamentous (sporophytes) were cultured in bubbling natural seawater with PES under 20 μmol photons m^–2^⋅s^–1^ at 20 ± 1°C and a 12:12 light:dark (L:D) photoperiod. HT as paternal was crossed to RZ as maternal by the methods by Niwa (2010) to generate the F1 offspring with sectored blades (chimeras) consisting of 1–4 sectors. Segregation of WT and red-type (RT) phenotypes in the F1 segregating populations was analyzed using chi-square (χ^2^) tests for goodness of fit (Sokal and Rohlf, 1995).

For each sector of four-sectored chimeras and the polar sector of three-sectored chimeras, all cells of each sector have the same genotype. Therefore, we directly used single-genotype sectors for the subsequent genetic analysis.

### Pigment Quantification

For pigment content analysis between RZ and HT, blades from two parents with the same living conditions were sampled, samples were frozen at −80°C before pigment extraction, and the contents of phycoerythrin (PE), phycocyanin (PC), allophycocyanin (APC), and chlorophyll *a* (*Chl*a) were subsequently measured. For *Chl*a, about 0.1 g of each sample was ground in 5 mL of 95% ethanol and extracted at 4°C in darkness for 24 h. For PE and PC, 0.1 g of each sample was ground in 10 mL of extraction buffer (0.1 M phosphate buffer, pH 6.8) at 4°C. The extract was centrifuged at 5,000 *g* for 10 min and then used to determine the contents of *Chl*a, PE, PC, and APC using an ultraviolet spectrophotometer (UV-180, Shimadzu, Japan). *Chl*a content was analyzed according to Wellburn (1994) and PE, PC, and APC contents were analyzed according to Kursar and Alberte (1983). Pigment measurement was conducted using three independent experimental repeats. All of these measurement operations were conducted in darkness to avoid degradation of photosynthetic pigments.

### DNA Isolation

Total DNA from single-genotype color sectors or parents’ blades was extracted using a Plant Genomic DNA Kit (Tiangen, China) following the manufacturer’s protocol. DNA concentration and quality were examined with the Qubit.2.0 fluorometer (Invitrogen) and 1% agarose gel electrophoresis and a N60 UV-Vis spectrophotometer (Implen, Munchen, Germany). DNA was subsequently stored at −80°C until further analysis.

### QTL-seq

The F1 single-genotype color sectors mentioned earlier were used in QTL-seq. A high-quality reference genome consisting of three chromosomes was from RZ line blades’ material (maternal). A total of five DNA pools were further constructed, namely, P1, RT24, WT24, RT56, and WT56. The P1 pool was from the blades of HT (paternal); two pairs of mixed pools RT24-WT24 and RT56-WT56 were constructed by equally mixing 24 or 56 F1 single-genotype sectors with different color traits (WT or RT). Paired-end sequencing libraries with insert sizes of approximately 350 bp and a read length of 150 bp were subjected to whole-genome resequencing with Illumina HiSeq 2000 platform (Illumina, United States).

The raw reads obtained from both parents and four progeny pools were filtered and aligned to the *P. yezoensis* genome sequence using the Burrows-Wheeler alignment tool (BWA) (Langmead and Salzberg, 2012). Picard software^1^ was used to mark duplicates. SAMtools (settings: -bS -t, “rmdup”) and GATK software were used to detect single-nucleotide polymorphisms (SNPs) and InDels (Li et al., 2009; McKenna et al., 2010). ANNOVER software was used to annotate SNPs or InDels based on the GFF3 files for the reference genome (Wang K. et al., 2010). SNPs/InDels filter standard pipelines were as follows: read depth ≥ 4, ≤ 1000; mapping quality ≥ 20; adjacent SNP distance ≥ 5 bp.

Homozygous SNPs/InDels between two parents were extracted from the VCF files. Using the RZ genome as reference, we calculated the statistic read number for this reference parent in the offspring pool. Then, SNP/InDel-index and the Δ(SNP/InDel-index) values were calculated to identify candidate genomic regions associated with the red-type trait of HT (DePristo et al., 2011). The Δ(SNP/InDel-index) was determined based on the difference in the SNP/InDel-index between the RT and WT pools. An average of Δ(SNP/InDel-index) of SNPs located in the given genomic interval was calculated using the sliding window approach with a 1-Mb window size and 100-kb increments. The Δ(SNP/InDel-index) of the RT and WT pools and their corresponding SNP/InDel-indices within the specified window size were plotted in a graph to generate SNP/InDel-index plots. The Δ(SNP/InDel-index) value should be significantly different from 0 if a genomic region harbors the target gene (Takagi et al., 2013). We calculated statistical confidence intervals of Δ(SNP/InDel-index) for all the SNP positions with given read depths and obtained 95% and 99% confidence intervals. By examining the Δ(SNP/InDel-index), the plot peak regions above the confidence value were defined as candidate regions for association with red coloration.

### Linkage Map-Based QTL Analysis

In order to confirm the accuracy of candidate region identified by QTL-seq, another 84 single-genotype color sectors from the F1 offspring were used to conduct linkage map-based QTL analysis. Markers for the construction of the genetic map were screened using the same protocol earlier described and stricter filter criteria (genotype deletion rate ≤ 0.2, MAF (minimum allele frequency) ≥ 0.05). QTL mapping was analyzed using R/qtl software (Arends et al., 2010). Interval mapping and composition interval mapping were conducted using the scanone() and cim() functions in R/qtl, respectively. The mapping step was set to 1 cM, and other parameters were set to the default value. To determine the threshold of logarithm of odd (LOD) scores, 1,000 permutations were conducted, and the threshold was selected at a 5% confidence level. Consecutive regions with LOD values larger than the threshold were called QTLs. For each QTL, the region with the highest LOD value was judged as the peak of this QTL. If the distance between peaks of two adjacent QTLs was <10 cM, then the QTLs were merged by the define.peak() function in the R package eqtl^2^. The boundary of each QTL was determined by 1.5-LOD drop support intervals. The regional genes were annotated and analyzed on the base of previous analysis of *P. yezoensis* in our lab.

### Narrowing Down the QTL Interval for Red Coloration

Using the information from the QTL-seq analysis of SNPs related to the candidate region, selected SNPs were confirmed using the Kompetitive Allele Specific PCR (KASP) method following the manufacturer’s instructions (Semagn et al., 2014). A total of 10 KASP markers were designed to genotype 328 individuals, including 8 from parents and 320 from single-genotype sectors of F1.

### RNA Sampling and RNA-seq

For gene expression analysis of the candidate region, blades from two parents living in the same condition were sampled for RNA-seq. Each parent sampling was conducted using three biological replicates. Total RNA was extracted using a Plant RNA Kit (OMEGA), and the first-strand cDNA was prepared using a HiScript II Q RT SuperMix for the qPCR kit (Vazyme Biotech). RNA quality was assessed using an RNA Nano 6000 Assay Kit and Bioanalyzer 2100 system (Agilent Technologies). Sequencing libraries were generated using a VAHTS Total RNA-Seq Library Preparation Kit (Vazyme Biotech). Then, the libraries were paired-end sequenced on an Illumina HiSeq 2000 platform (Illumina, United States) with the read length of 150 bp.

The quality of raw sequencing reads was evaluated using FastQC (Brown et al., 2017) and Trimmomatic (Bolger et al., 2014). Differential gene and transcript expression analysis of RNA-seq experiments were performed with TopHat and Cufflinks, respectively (Trapnell et al., 2012).

### Sequence Analysis and Prediction of the Candidate Gene

The *P. yezoensis* sequence and gene functional annotations were deposited to DDBJ/ENA/GenBank as accession WMLA00000000. Conserved domains were identified in NCBI^3^ and Pfam^4^ databases. Amino acid sequences were aligned using MEGA 7 (Kumar et al., 2016) and ClustalX 2.0 (Larkin et al., 2007).

### Quantitative RT-PCR

RNA sampling and cDNA synthesis were as earlier described. Three biological and technical replicates were used for qRT-PCR analysis. Quantitative RT-PCR (qRT-PCR) was conducted using a ChamQ SYBR color qPCR Master Mix (Vazyme Biotech). The primers for qRT-PCR were designed using Primer 5.0 software. And the selected genes were verified using the Light-Cycle^®^ 480 Real-Time PCR System with the following cycling conditions: 95°C for 90 s, followed by 40 cycles of 95°C for 5 s, 60°C for 15 s and 72°C for 20 s. The ubiquitin conjugating enzyme (UBC) gene was used as reference gene. The sequences of the primers used are listed in Supplementary Table S10. The 2^–ΔΔCt^ method was used to calculate relative gene expression values.

### Phylogenetic Analysis

Phylogenetic neighbor-joining (NJ) trees were constructed using MEGA 7 software using the bootstrap method and 1,000 replications. The phylogenetic relationships of the studied red algae were assessed using TimeTree v3.0^5^.

## Results

### Inheritance Pattern of the Phenotype of Red Coloration

After crossing RZ and HT (Figure 1), carpospores were brushed from the maternal RZ blades. The carpospores were cultivated individually, which developed into conchocelis clones and then induced to release conchospores separately. For color sectors statistical analysis, only the F1 gametophytes with chimeric coloration were selected to exclude the blades generated from the self-fertilized carpospores. The chimeric gametophyte blades indicated that the carpospores and their conchocelis were heterozygous when generated from cross-fertilization between the two strains.

The color phenotypes and blade types of F1 gametophytic blades from the heterozygous conchocelis in the cross between RZ and HT were counted (Supplementary Table S1). Eight types of blades, including two types of unsectored blades and six types of sectored blades with 2–4 sectors, were observed in the F1 gametophytic blades (Supplementary Table S1), and only the two parental color types were observed in the offspring, including unsectored and sectored blades (Figure 1E). The frequency of sectored F1 blades was higher than the frequency of unsectored F1 blades. Statistical analysis revealed that the segregation ratio of the WT sectors and RT sectors in the F1 sectored blades was approximately 1:1 (χ^2^ = 1.90 < χ_0.05_^2^ = 3.84; Table 1). The results suggested that the inheritance pattern of red coloration trait was in accordance with Mendel’s laws, which could be identified as a qualitative trait controlled by a single nuclear allele.

**TABLE 1:** Segregation ratios of different color sectors in F1 gametophytic blades from heterozygous conchocelis in the cross between RZ and HT of *P. yezoensis*.

	Segregation ratio
WT:RT	5,830:5,682
	(1:0.97)

### Pigment Variations Between WT and Red Mutants

**FIGURE 2:**
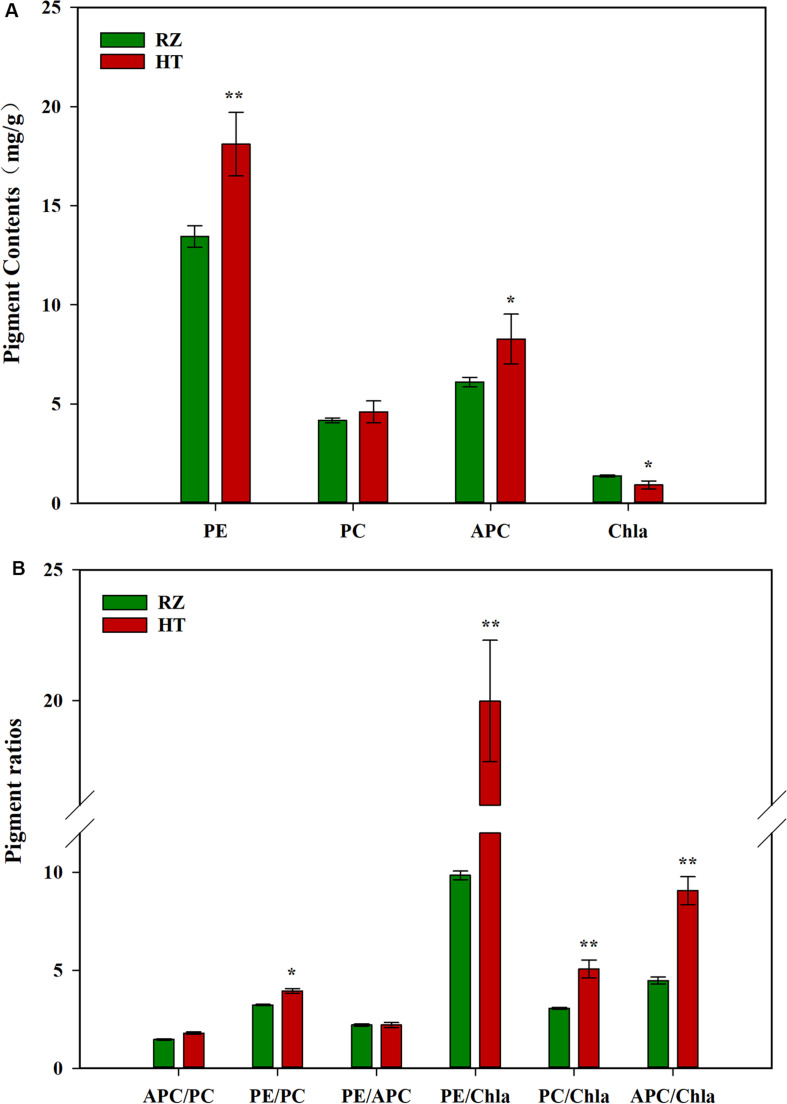
**(A)**: Photosynthetic pigment contents among the parents RZ and HT. PE, phycoerythrin; PC, phycocyanin; APC, allophycocyanin; *Chla*, chlorophyll *a*. **(B)** Pigment content ratios in the gametophytic blades of RZ and HT.

Pigment contents varied between the two parents (RZ and HT, Figure 2A), which were cultured under the same conditions for the same duration. RZ possessed significantly greater concentrations of *Chl*a than HT (*P* < 0.05). PE and APC concentrations in HT were, respectively, 34.53% (*P* < 0.01) and 35.41% (*P* < 0.05) higher than RZ. However, PC concentrations in HT were 10.29% higher than RZ, which were not significantly different (*P* > 0.05). The ratios of pigments in blades of the RZ and the HT were also compared (Figure 2B). These three ratios significantly differed between the mutant and RZ: The PE/*Chl*a, PC/*Chl*a, APC/*Chl*a, and PE/PC of HT were significantly higher than RZ (*P* < 0.05). However, no significant differences in the APC/PC and PE/APC between HT and RZ were observed (*P* > 0.05).

### Mapping of the Genetic Locus of *rcl-1*

**FIGURE 3:**
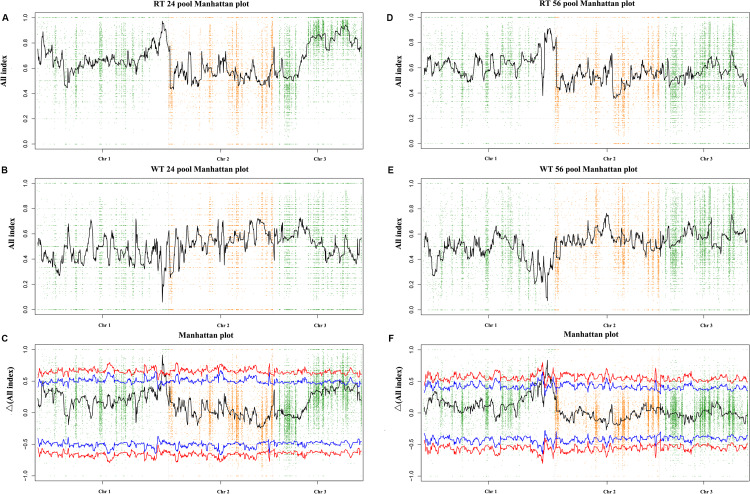
Δ(All-index) graphs from QTL-seq analysis. The Δ(All-index) plot with statistical confidence intervals under the null hypothesis of no QTL (red, *P* < 0.01; blue, *P* < 0.05). The *X*-axis represents the position of 3 chromosomes, and the *Y*-axis represents the Δ(All-index). The Δ(All-index) was calculated based on a 1-Mb interval with a 100-kb sliding window. **(A–C)**: RT24-WT24, and **(D–F)**: RT56-WT56.

By resequencing the five genomic libraries from the pools, including the HT and four progeny mixed pools, a total of 133.4 Gb was generated with an average genome sequencing depth of 1,243.5× (Supplementary Table S2). The sequence reads of the five DNA pools were aligned to the reference genome, and a batch of SNPs loci and InDels was found. A total of 24,159 SNP/InDels that occurred in HT and not in RZ were considered in calculating the SNP/InDel-index (All-index). The Δ(All-index) was calculated based on the All-index of the WT pools and RT pools (Figure 3). The Δ(All-index) value should be significantly different from 0 if a genomic region is harboring the target gene. For the RT24-WT24 pools, four regions were significantly different from 0, which spanned a total of 7.9 Mb (Table 2) at the 95% significance level; for RT56-WT56 pools, only two regions at the end of chromosome 1 were significantly different from 0, which spanned a total of 4.9 Mb (Table 2). These results indicate that the overlapping region of the two-pair locating was the candidate region harboring the red coloration locus, which was from 39,700,001 to 43,000,000 on chromosome 1 (Table 2).

**TABLE 2:** Candidate regions related with red coloration identified by QTL-seq.

Pool-pair	Candidate regions	Overlapping region
	(95% significance level)	
RT24-WT24	1,400,001–2,700,000 on chromosome 1	39,700,001–
	39,600,001–43,200,000 on chromosome 1	43,000,000 on
	21,900,001–23,900,000 on chromosome 3	chromosome 1
	27,000,001–28,000,000 on chromosome 3	
RT56-WT56	36,400,001–38,000,000 on chromosome 1	
	39,700,001–43,000,000 on chromosome 1	

A total of 84 single-genotype color sectors from RZ × HT were used to confirm the region 39,700,001–43,000,000 on chromosome 1. A total of 17,888 markers were screened to construct a genetic map, then cosegregating markers were clustered in recombination bin markers, and finally, a total of 243 bin makers were used to construct the genetic map (Supplementary Figure S1). The map consisted of 3 chromosomes and covered 472.45 cM, with an average distance of 1.94 cM (Supplementary Table S3). QTL analysis of RT and WT traits was performed using the R/qtl software packages to produce consensus QTL regions. QTL region between Marker4105 and Marker4115 located near candidate region was identified in chromosome 1 (Supplementary Figure S1). Marker4105 and Marker4115 were 3.57 cM apart, corresponding to physical distance of 2.74 Mb (region 40,839,807–43,583,607) on chromosome 1, based on the *P. yezoensis* genome sequence, which were overlapping with region identified by QTL-seq. This region through QTL-seq was also identified in the results of linkage map-based QTL analysis and was thus referred to as the candidate region harboring a causal locus for red thallus coloration.

A total of 36 SNP sites at physical position 39.70–43.00 Mb of chromosome 1 were identified by QTL-seq. To further narrow down the region, we developed 10 KASP markers, and finally, four valid KASP markers in this region were used for genotyping an additional set with 328 samples (Supplementary Table S4). Recombination events were detected in nine samples (F1-PWT14-2, F1-PWT2-2, F1-W12-1, F1-W21-3, F1-W22-1, F1-WT16, F1-WT33, F1-WT55, and F1-WT81) at the SNP1 locus. However, no recombination events were found in the other three loci due to high cosegregating strength. SNP1 is located at site 41,578,129 of the assembled genome. Then the candidate region was narrowed down to 1.42 Mb (41,578,129–43,000,000 on chromosome 1). Using the RZ genome as reference, there were 141 genes annotated in this candidate region. Among the 27 SNPs identified in the final candidate region (Supplementary Table S5), 24 SNPs were located within the intergenic region; two SNPs were located in up/down regulatory region that was associated with *Py04887*, *Py08429*, and *Py08430*. Subsequent transcriptome and qRT-PCR analysis revealed that *Py04887* had no expression and *Py08430* did not exhibit significant changes in RZ and HT (Supplementary Figure S5, *P* > 0.05). Remarkably, only one non-synonymous SNP [A/C] in the coding region of *Py08429* was located in the final candidate region.

### Expression Analysis and Candidate Gene Screening

To investigate the genes involved in red coloration in laver, samples collected from two parents were subjected to RNA-seq analysis (Supplementary Table S6). A total of 1,459 differential expressed genes were detected (|log_2_(fold_change)| >1), including 605 annotated genes and 854 unannotated genes (Supplementary Table S7; Supplementary Figure S2). Compared with RZ, 718 genes were upregulated, and 741 genes were downregulated in HT. To validate the expression profiles obtained by RNA-seq, qRT-PCR was performed on ten genes in this study. For all of the ten genes, the trends in qRT-PCR differential expression (*P* < 0.05) were in agreement with the RNA-seq differentially expressed analysis except for *Py04880*, *Py08094*, and *Py06313* (Supplementary Figure S6), which means the results of transcriptional analysis were reliable.

Among these genes, one gene, which is predicted to be the gamma subunit of phycoerythrin (*Py09239*) and directly associated with pigments, was upregulated in HT compared to RZ; this finding coincides with the pigment content results; while in the chlorophyll metabolic pathway, the expression level of magnesium chelatase subunit H (*Py02527*) and geranylgeranyl reductase (*ChlP*, *Py03856*) decreased in HT compared to RZ; although these three genes were not included in the candidate region described above.

**FIGURE 4:**
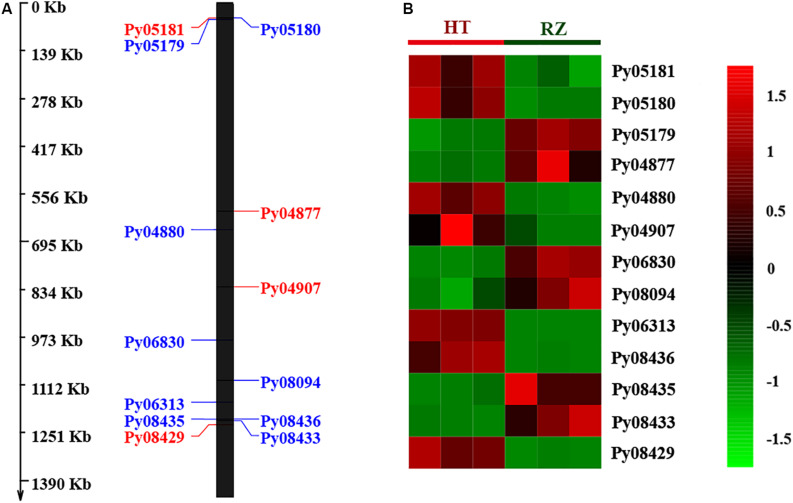
**(A)** Distribution of 13 differential expressed genes (DEGs) in candidate region. Red, genes on the positive strand. Blue, genes on the negative strand. **(B)** Heat map of 13 DEG expression patterns in Blades of RZ and HT.

**FIGURE 5:**
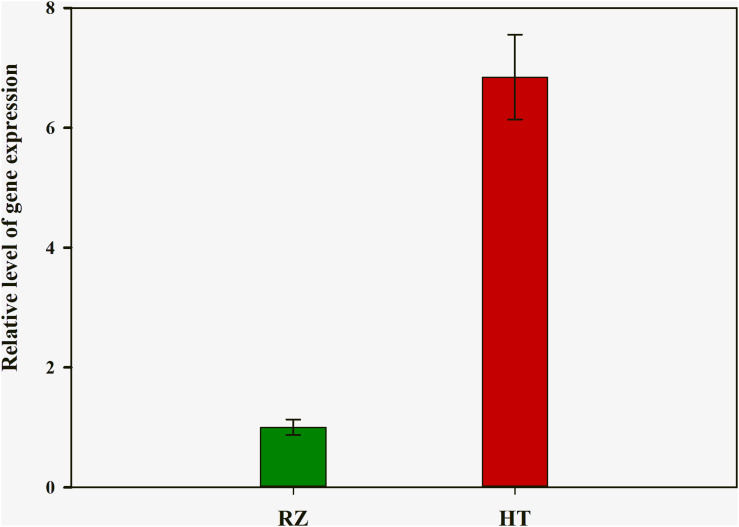
Quantitative real-time polymerase chain reaction (PCR) analysis of *rcl-1* among the wild-type (RZ) and the red mutant (HT). The mean ± SD of the three biological replicates is presented.

To delineate the potential candidate gene regulating red coloration in RZ and HT, differential expression profiling of 141 genes predicted within the 1.4-Mb candidate region was performed based on the transcriptome data above. Thirteen genes showed significant differences in expression between RZ and HT (|log_2_(fold_change)| >1) (Figure 4; Supplementary Table S8). Combined with the locations of SNPs at candidate region, one SNP (A/C)-carrying *Py08429* gene exhibiting pronounced upregulation (>6-folds) in red mutant (HT) (Figures 4B, 5) was considered as the candidate allele regulating red coloration in *P. yezoensis*.

We cloned *Py08429* from both RZ and HT. The results suggested that the presence of one exon and the predicted coding sequence (CDS) of the cloned *Py08429* cDNA was 1,338 bp, with a predicted corresponding protein length of 446 amino acids. Alignment of the *Py08429* sequence between the RZ and HT revealed a single non-synonymous A→C mutant in the exon, resulting in the conversion of a glutamine (Gln, Q) to proline (Pro, P) at residue 331. Searching in the conserved domain database (CDD) and BLAST in NCBI did not reveal any annotation information on *Py08429*.

To investigate the conservation of the red mutant locus, 21 genetically distinct wild-type *P. yezoensis* lines were selected, and these *Py08429* sequences were determined. All these WT *P. yezoensis* lines shared the RZ protein sequence in this site (Supplementary Figure S3). These findings indicated that *Py08429* is the primary candidate for red coloration locus, and we named it *rcl-1*.

### Phylogenic Analysis

To better predict the information of *rcl-1*, we searched the public database NCBI and 18 published genome databases from a range of plant species (Supplementary Table S9) using BLAST with the *rcl-1* amino acid sequence. Only red algae (*Pyropia haitanensis*, *Porphyra umbilicalis*, *Porphyridium purpureum*, *Galdieria sulphuraria*, and *Cyanidioschyzon merolae*) showed *rcl-1* homologs.

**FIGURE 6:**
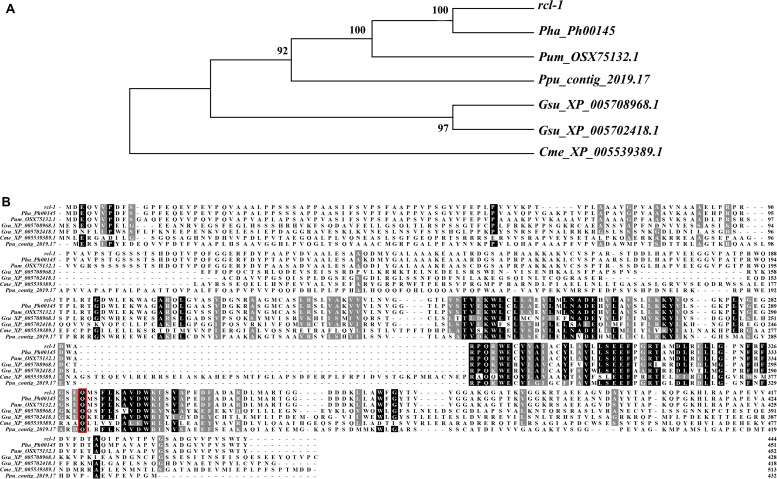
**(A)** Phylogenetic analysis of *rcl-1* and its homologs in four other red algae. Evolutionary relationships were inferred using the neighbor-joining method. The bootstrap test values (1,000 replicates) are shown next to the branches. **(B)** Amino acid alignment of *rcl-1*proteins in five red algae genomes. Red boxes: mutation sites.

Sequence alignment revealed that the protein shares 93.36% sequence identity with *Pha_Ph00145*, 84.73% sequence identity with *Pum_OSX75132.1*, 42.21% sequence identity with *Ppu_contig_2019.17*, 42.54 and 37.89% sequence identity with *Gsu_XP_005702418.1* and *Gsu_XP_005708968.1*, respectively, and 27.88% sequence identity with *Cme_XP_005539389.1*. The site of *rcl-1* protein where the non-synonymous mutation occurred is highly conserved (Figure 6B).

To further investigate the relationship between *rcl-1* and other homologs, we used MEGA 7 to build an NJ tree for these proteins (Figure 6A). The phylogenic pattern of these genes was the same as the topology of the species evolutionary tree (Supplementary Figure S4).

## Discussion

### Populations and Methods Used for the QTL-Mapping in *Pyropia*

In this study, we used red mutant and QTL-seq-based combined methods to fine map a red coloration locus (*rcl-1*). The life history and generation alteration in *Pyropia* are quite different from those of higher plants (Niwa, 2010); therefore, the commonly used protocols for the construction of the genetic-mapping population were not suitable for the *Pyropia*. *Pyropia*’s life history includes sexual reproduction and asexual reproduction. In sexual reproduction, the blades of *Pyropia* are developed from the conchospores (2n) released from filaments propagated from carpospores. During the germination of the conchospores, meiosis occurs and the haploid, ordered tetrads are generated (Ohme and Miura, 1988). It is different from the formation of individual germ cells by meiosis in animals and higher plants that the four cells [ordered tetrad (n)] produced by the meiosis in *Pyropia* are joined together, and thus the genotype of each cell in the tetrad could be different due to homologous recombination during meiosis (Ohme and Miura, 1988). Each cell (n) of the ordered tetrad sequentially develops into a part of the blade by mitosis, and thus the blade of the laver is a genotype chimeric haploid (except for those developing from homozygous conchospores). Generally, the single-genotype sectors of chimeric blades cannot be visually distinguished (Yan and Aruga, 2000; Shimizu et al., 2007). Asexual reproduction of the laver blades results in the release of a large number of monospores that grow into blades, thereby providing large amounts of materials that were used in parallel repeated measurements of the single-genotype trait at one time (Chen et al., 2019).

With the help of the color mutants, the four cells of the tetrad with different genotypes generated from the homologous recombination during meiosis can be clearly distinguished, which are suitable materials for the genetic mapping (Xu et al., 2015; Huang and Yan, 2019a). Previous studies have reported that laver coloration mutants controlled by single nuclear genes were used for genetic traits location (Niwa et al., 2002; Niwa, 2010). Single-color sectors have small biomass and incomplete leaves, which limit trait measurement and sequencing. To next obtain enough materials for trait measurement and DNA sequencing, previous research works provide a protocol by constructing a stable permanent mapping population (DH population) to obtain enough materials (Xu et al., 2015; Huang and Yan, 2019a, b). Here, we propose a simpler and time-saving method for the construction of the mapping population of any trait location compared to the protocol of constructing a DH population. After obtaining a large amount of color-sectored blades through a cross between the coloration mutant and the WT, the distinguished single-genotype color sectors were sectioned into small pieces individually to induce the monospores, and the monospores from one single-genotype color sector germinate into a gametophytic population with the same genotype.

In this experiment, we performed hybridization to obtain hybrid filaments and released number conchospores to obtain several progeny chimeric blades. Statistical analysis of the number of offspring color sectors proved that the color trait of the red mutant in this experiment was in accordance with Mendel’s laws, indicating that this trait is controlled by a single nuclear gene. Using the life history of *P. yezoensis* and the advantages provided by pigment mutants, we obtained several single-genotype and single-color sectors. Because the color trait of the red mutant in this study is a qualitative trait, we can determine the color trait during the chimera period. Therefore, we opted to directly use these single-genotype sectors with different single-color traits (RW or WT) for pooling. As for other qualitative and quantitative traits that need to be determined by measuring intact individuals such as length and width, it is still necessary to induce monospores and culture these into intact leaves for further genetic analysis. The advantages of QTL-seq vis-a-vis other available traditional QTL mapping approaches to identify major QTLs governing fruit and leaf color in higher plants (including *A. thaliana, Brassica rapa*, and pear) have recently been reported (Wang et al., 2016; Hsieh et al., 2017; Xue et al., 2017). The number of individuals contained in a mixed pool also influences the positioning accuracy. By comparing the candidate areas determined by two pairs of mixing progeny pools, the pool-pair containing 56 sectors has higher positioning accuracy and smaller positioning range. Although this study used two pairs of pool data to identify a single candidate interval, in the future, we can use only one mixed pool with a larger number of individuals and further narrow the interval using the next KASP (Gu et al., 2017). Compared with the positioning effect of KASP in higher plants (Kerbiriou et al., 2016; Qureshi et al., 2018), the positioning interval of this study is slightly larger than some higher plants, and this may be due to the strong linkage between mutations. However, it cannot be denied that this method is effective and efficient here, reducing the candidate region from 3.30 Mb to 1.42 Mb. The large-scale increase in the number of KASP-localized individuals may have an impact on location improvement, but the improvement may not be substantial. Therefore, we used RNA-seq to identify differentially expressed genes in candidate regions and further combined the results of mutation sites to identify the candidate gene.

Based on the unique characteristics of the life history of laver, we propose a strategy for studying genetic traits using hybridization with pigment mutants to obtain a large number of single genotype color sectors (to obtain numerous single-genotype leaves by releasing monospores), evaluating traits, and then using adjusted QTL-seq, and combined KASP and RNA-seq methods for trait location. Members of Bangiales have similar life cycle and characteristics, and this study has demonstrated the effectiveness of this strategy and provided ideas for other traits controlled by single nuclear-encoded genes or some threshold traits in Bangiales and even algae.

### Genetic Loci Related to Plant Coloration

Blade color mutants have been extensively studied in the past years. So far, almost 100 blade color genes have been cloned in higher plants. These causal genes have directly or indirectly changed the pigment contents of color mutants. The *SiYGL1* gene encoding a magnesium-chelatase D subunit (*CHLD*), *OsYGL1* gene encoding the chlorophyll (*Chl*) synthase, and *ZmYGL1* predicted to encode a cpSRP43 protein are associated with chlorophyll biosynthesis, and mutations in these genes result in *Chl*-defective yellow-green leaf mutants (Wu et al., 2007; Guan et al., 2016; Li et al., 2016). *OsSGR*, which encodes an ancient protein containing a putative chloroplast transit peptide, is related to the *Chl*-degrading pathway. The upregulation of SGR increases *Chl* breakdown during senescence (Jiang et al., 2007). The *OsPDS* gene that encodes a phytoene desaturase (PDS) is essential to the synthesis of carotenoid precursors of abscisic acid (ABA), and transgenic rice plants harboring the PDS-RNAi construct exhibit an albino phenotype (Miki and Shimamoto, 2004). In addition to direct causal genes, many genes indirectly interfere with the metabolism of pigments through multiple biological pathways such as *FvRAP* that encodes a GST anthocyanin transporter, *AtCPSAR1* that encodes a GTP-binding protein, and *AtGNC* (GATA nitrate-inducible carbon-metabolism-involved) and *AtCGA1* (cytokinin-responsive gata1) (Garcia et al., 2010; Chiang et al., 2012; Luo et al., 2018). In the present study, we isolated a red-blade mutant HT and mapped *rcl-1* that controls red coloration in HT to chromosome 1, which encodes a protein containing 446 amino acid residues. CDD and BLAST queries in NCBI did not reveal any annotation information on this gene, and this unannotated phenomenon is common in red algae (Nozaki et al., 2007; Brawley et al., 2017).

Unlike pigment components in higher plants, the gametophytic blade of *Pyroipa* usually contains three major pigments, namely, phycoerythrin, phycocyanin, and chlorophyll *a* (Zhang et al., 2014). Blade color varies with the content and proportion of these pigments which can determine the quality of the commercial dried blades ‘hoshi-nori’ in the nori industry (Yan et al., 2000). Yan et al. (2000) reported that the different phenotypic colors of three pigment mutants reflected the quantitative variations of phycobilins (*Chl*a, PE and PC), the pink mutant with high PE/*Chl*a and PE/PC ratios. Here, we found that the content of *Chl*a in HT was significantly lower than RZ blades, and among the pigment ratios, the PE/*Chl*a, PC/*Chl*a and APC/*Chl*a of HT was extremely significantly higher than RZ. These results reveal that the observed color change is mainly caused by a decrease in *Chl*a content. RNA-seq also show that the expression level of magnesium chelatase subunit H and the geranylgeranyl reductase (*ChlP*) gene significantly decreased in HT compared to RZ, which could directly decrease the level of *Chl*a content in RZ. In addition, we found that the content of PE in HT was significantly upregulated compared to RZ, and the expression level of phycoerythrin subunit in HT was higher than RZ, which coincided with the increase in pigment content. Some investigations have found that the decrease in the contents of chlorophyll and total carotenoids can be accompanied by an increase in phycoerythrin abundance of phycobilisomes (Shpilyov et al., 2005). Some genes such as protoporphyrin IX in the porphyrin and chlorophyll metabolism pathway are also related to the synthesis of biliverdin, which is a precursor of phycoerythrin (Huang et al., 2017). This phenomenon reveals that there are some genes which are competitive in both synthesis pathways. We thus infer that *rcl-1* may be related to the *Chl*a metabolic pathways, which results in a decrease in *Chl*a content and in turn increasing PE content, thereby leading to color changes. However, it is unclear if this qualitative gene that controls the coloration of the red-mutant blades is derived from the structural gene or transcription factor gene of the pigment biosynthetic pathway. Therefore, it is not possible to exclude the presence of *rcl-1* that acts as a switch in the coloration of red-mutant blades upstream of pigment metabolic pathway-related genes or transcription factors.

By searching for *rcl-1*’s homologs among 18 published genome databases from a range of plant species, we found that only red algae contain *rcl-1* homologs. The high degree of protein sequence conservation with homologs from red algae species further suggests the importance of the *rcl-1* non-synonymous mutations. The gene associated with red coloration in *P. yezoensis* and its absence in higher plants and green algae also suggests that *rcl-1* may also be related to the unique pigment composition system and even the photosystem of red algae. The results of previous investigations and the present study clearly indicate the need for more detailed functional validation and detailed molecular characterization of this gene to understand its definite role in color regulation in *P. yezoensis*. Gene editing technology in *Pyropia* is currently in progress, and in the future, this technology may help us fully confirm and understand its function. Therefore, a red coloration *rcl-1* gene could be potentially utilized as a molecular marker for genetic analysis and marker-assisted genetic improvement of *Pyropia*.

## Conclusion

In this study, we identified the genetic locus that regulates the red coloration in *P. yezoensis.* First, genetic analysis showed that this trait followed Mendel’s laws, indicating that it is a qualitative trait. Then, we used a combination of QTL-seq and KASP to narrow down the region containing the *rcl-1* gene to a segment of 1.42 Mb in size. Combined with RNA-seq, *rcl-1*, which contains one non-synonymous SNP [A/C] in the coding region and different expression levels between RZ and HT, has been shown to be essential for regulating red coloration in *P. yezoensis*. This is the first report on fine mapping functional genes in red algae and the elucidation of the relationship between *rcl-1* genetic locus and pigment regulation.

## Data Availability Statement

The RNA-seq data for this study can be found in the National Center for Biotechnology Information Sequence Read Archive under the accession numbers SRR11820821–SRR11820826.

## Author Contributions

XY and YM designed the study. XY, LW, and BS conducted the experiments. XY and KX analyzed the data. XY wrote the manuscript, which was revised by YM, FK, DW, and XT. All authors read and approved the final version of the manuscript.

## Conflict of Interest

The authors declare that the research was conducted in the absence of any commercial or financial relationships that could be construed as a potential conflict of interest.

## Footnotes

1 https://sourceforge.net/projects/picard/

2 https://cran.r-project.org/web/packages/eqtl/index.html

3 https://www.ncbi.nlm.nih.gov/

4 http://pfam.xfam.org/

5 http://www.timetree.org/

## Supplementary Material

FIGURE S1 Traditional map-based QTL mapping of the *rcl-1* gene. 243 bin makers were selected between two parents, and genotyping in an DH population of 84 individuals delimited *rcl-1* to a 2.74 Mb region with flanking marker Marker4105 and Marker4115, respectively.

FIGURE S2 GO **(a)** and KEGG **(b)** enrichment of DEGs. The bubble diagram shows the degree of enrichment of GO and KEGG terms in three categories. By default, the top 20 GO terms with the lowest *Q*-values were used in the diagram. The *X*-axis represents the enrichment ratio, and the *Y*-axis denotes the GO term or KEGG pathway. The size of bubbles indicates the number of genes annotated to a certain GO term or KEGG pathway, and the color represents the *Q*-value, where the darker the color is, the smaller the *Q*-value is.

FIGURE S3 Multiple sequence alignment of the amino acid sequences of *rcl-1* proteins from 21 genetically distinct wild-type *P. yezoensis* lines, RZ and HT. (Black = Conversed region).

FIGURE S4 The topology of the species evolutionary tree.

FIGURE S5 Quantitative real-time polymerase chain reaction (qPCR) analysis of *Py08430* gene among the wild-type (RZ) and the red mutant (HT). The mean ± SD of the three biological replicates is presented.

FIGURE S6 Quantitative real-time polymerase chain reaction (qPCR) analysis of 10 genes among the wild-type (RZ) and the red mutant (HT). The mean ± SD of the three biological replicates is presented.

TABLE S1 Color phenotypes and blade types in F1 gametophytic blades from heterozygous conchocelis in the cross between the WT RZ and the red mutant HT of *Pyropia yezoensis*. W: wild type (WT); R: red type (RT).

TABLE S2 The statistics and evaluation of sequencing data generated from paternal parent and the extreme pools in QTL-seq.

TABLE S3 Statistics of linkage map developed in this study.

TABLE S4 Gene primers designed for KASP.

TABLE S5 SNPs in candidate region by QTL-seq and KASP.

TABLE S6 The statistics of sequencing data and genome comparison in RNA-seq.

TABLE S7 FPKM values and annotation information of differential expressed genes in RNA-seq.

TABLE S8 Differentially expressed genes in the candidate region of *P. yezoensis* chromosome 1.

TABLE S9 Protein databases in NCBI were used for searching homologs of *rcl-1*.

TABLE S10 Primers used in qRT-PCR for validating differentially expressed genes.
