# Supplementary figures and images for: Fine Mapping to Identify the Functional Genetic Locus for Red Coloration in Pyropia yezoensis Thallus
Source: Front Plant Sci. 2020 Jun 23;11:867. doi: 10.3389/fpls.2020.00867 (PMC7324768; doi:10.3389/fpls.2020.00867)

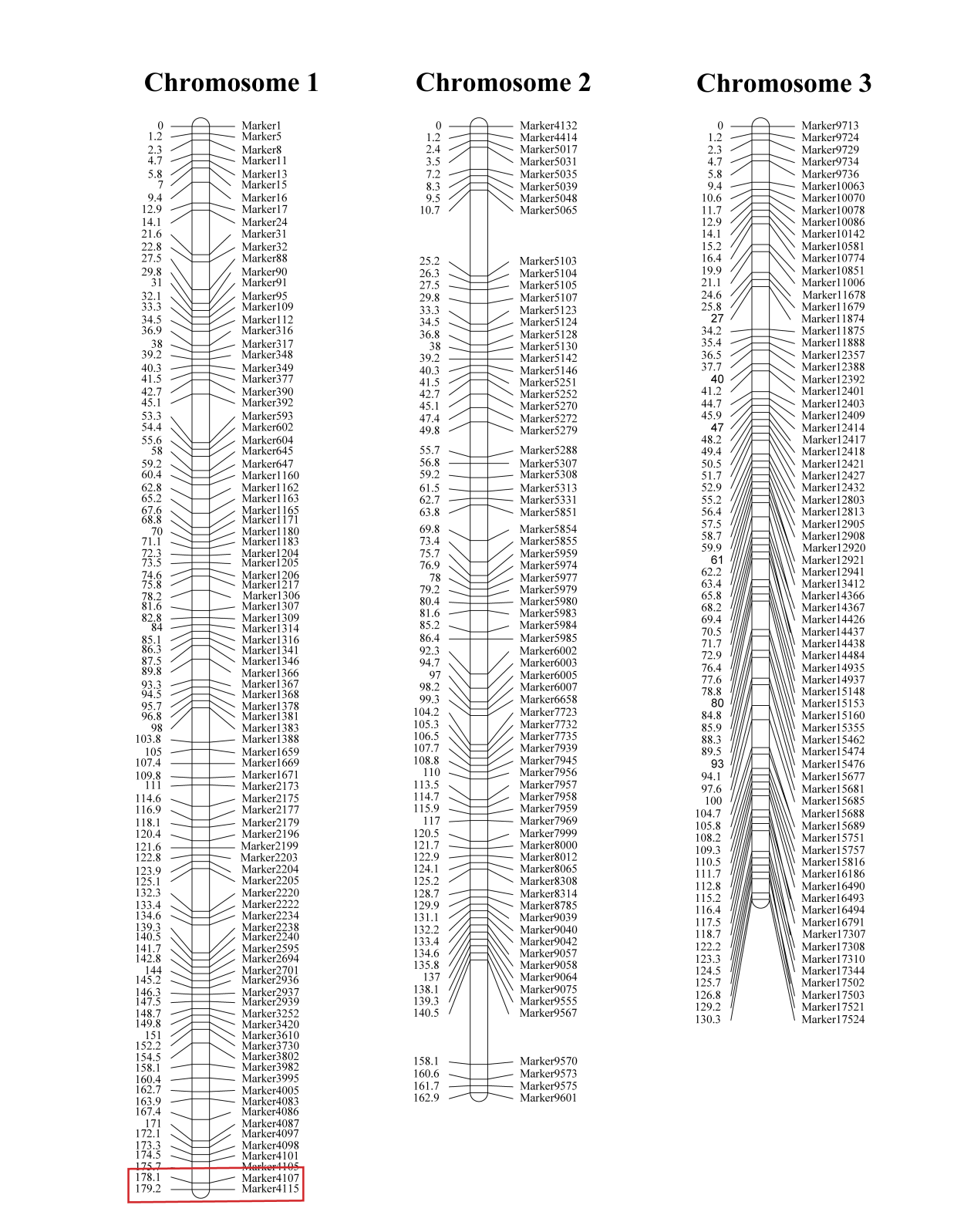

Supplement: FIGURE S1 — Traditional map-based QTL mapping of the rcl-1 gene. 243 bin makers were selected between two parents, and genotyping in an DH population of 84 individuals delimited rcl-1 to a 2.74 Mb region with flanking marker Marker4105 and Marker4115, respectively. [file Image_1.TIF]

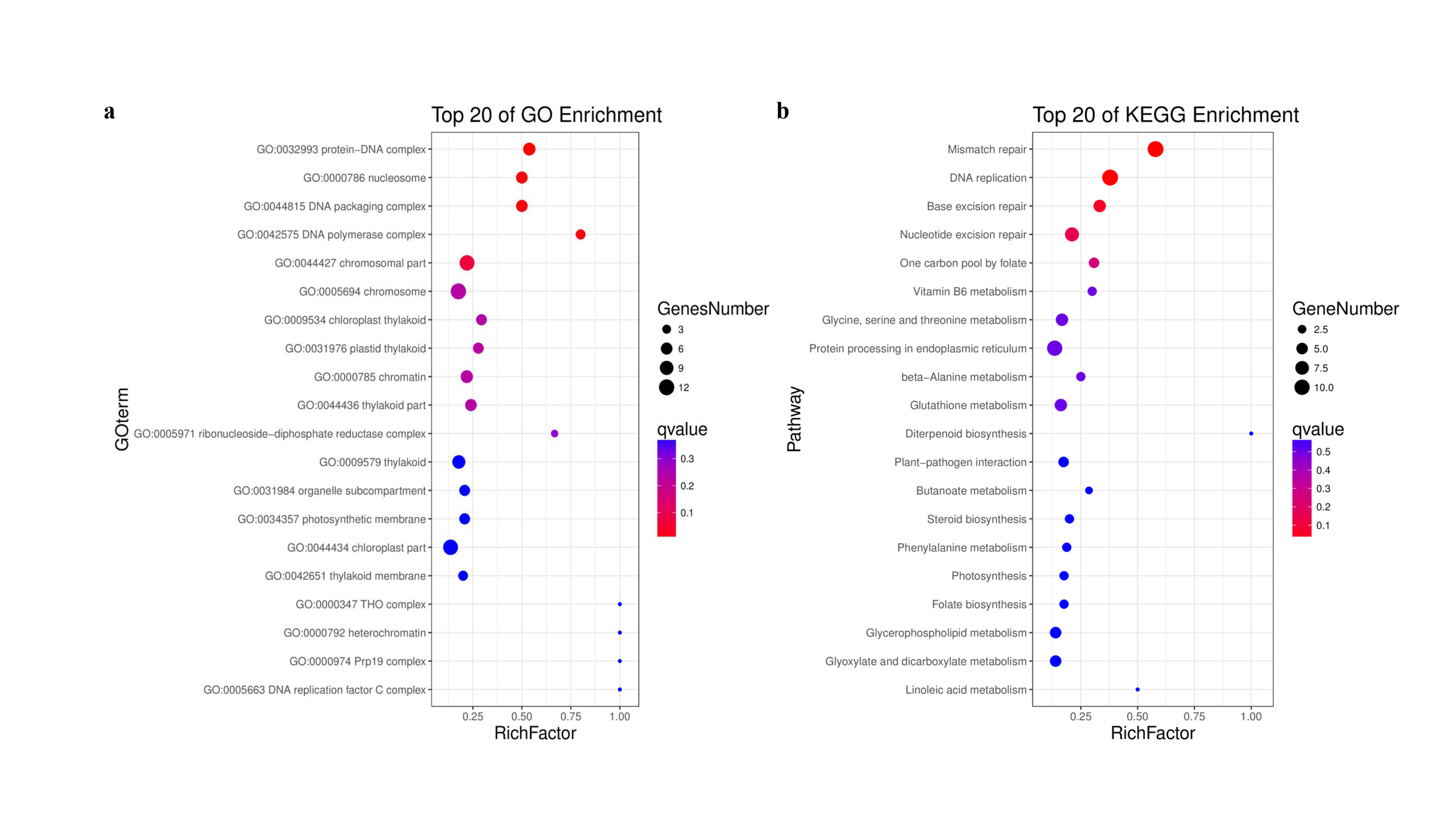

Supplement: FIGURE S2 — GO (a) and KEGG (b) enrichment of DEGs. The bubble diagram shows the degree of enrichment of GO and KEGG terms in three categories. By default, the top 20 GO terms with the lowest Q-values were used in the diagram. The X-axis represents the enrichment ratio, and the Y-axis denotes the GO term or KEGG pathway. The size of bubbles indicates the number of genes annotated to a certain GO term or KEGG pathway, and the color represents the Q-value, where the darker the color is, the smaller the Q-value is. [file Image_2.TIF]

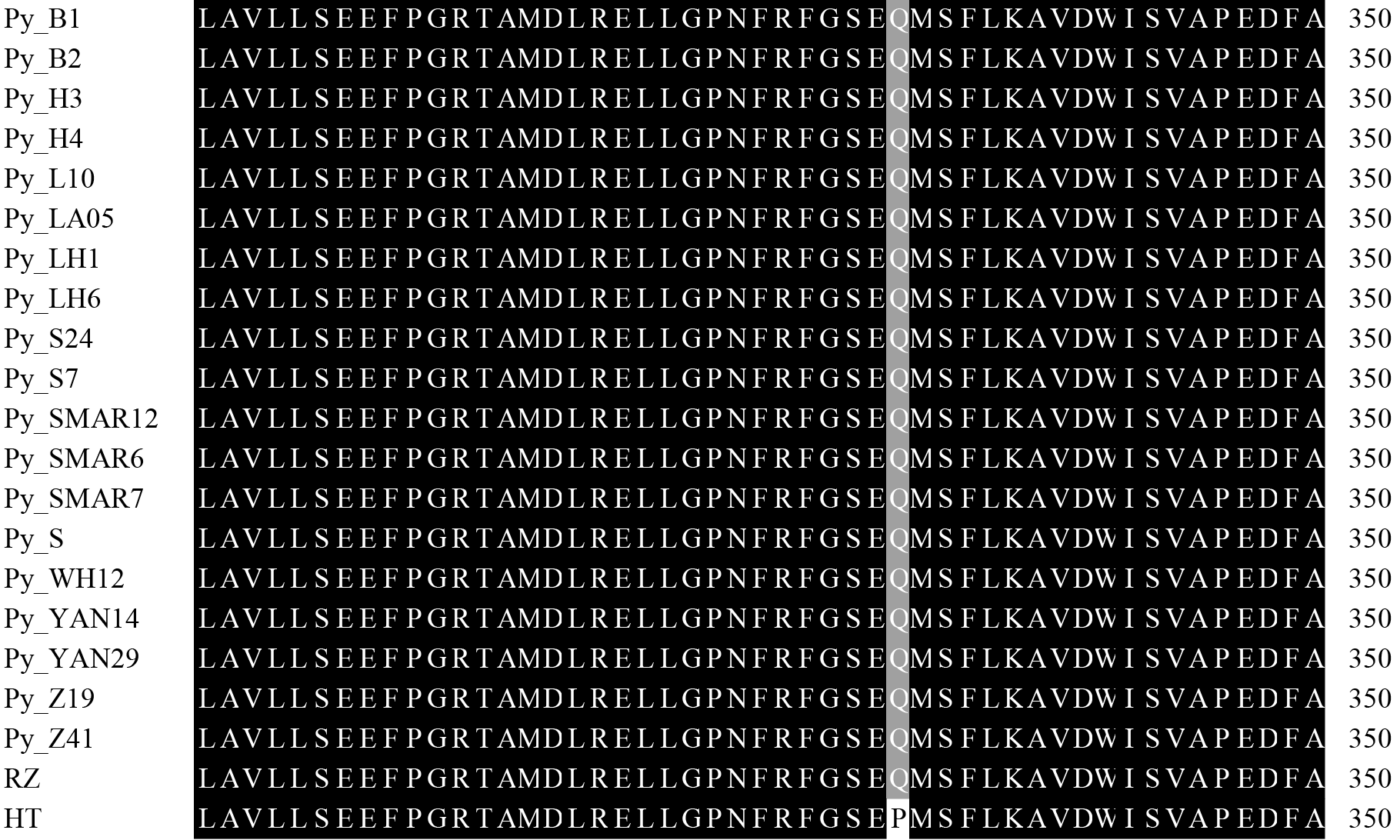

Supplement: FIGURE S3 — Multiple sequence alignment of the amino acid sequences of rcl-1 proteins from 21 genetically distinct wild-type P. yezoensis lines, RZ and HT. (Black = Conversed region). [file Image_3.TIF]

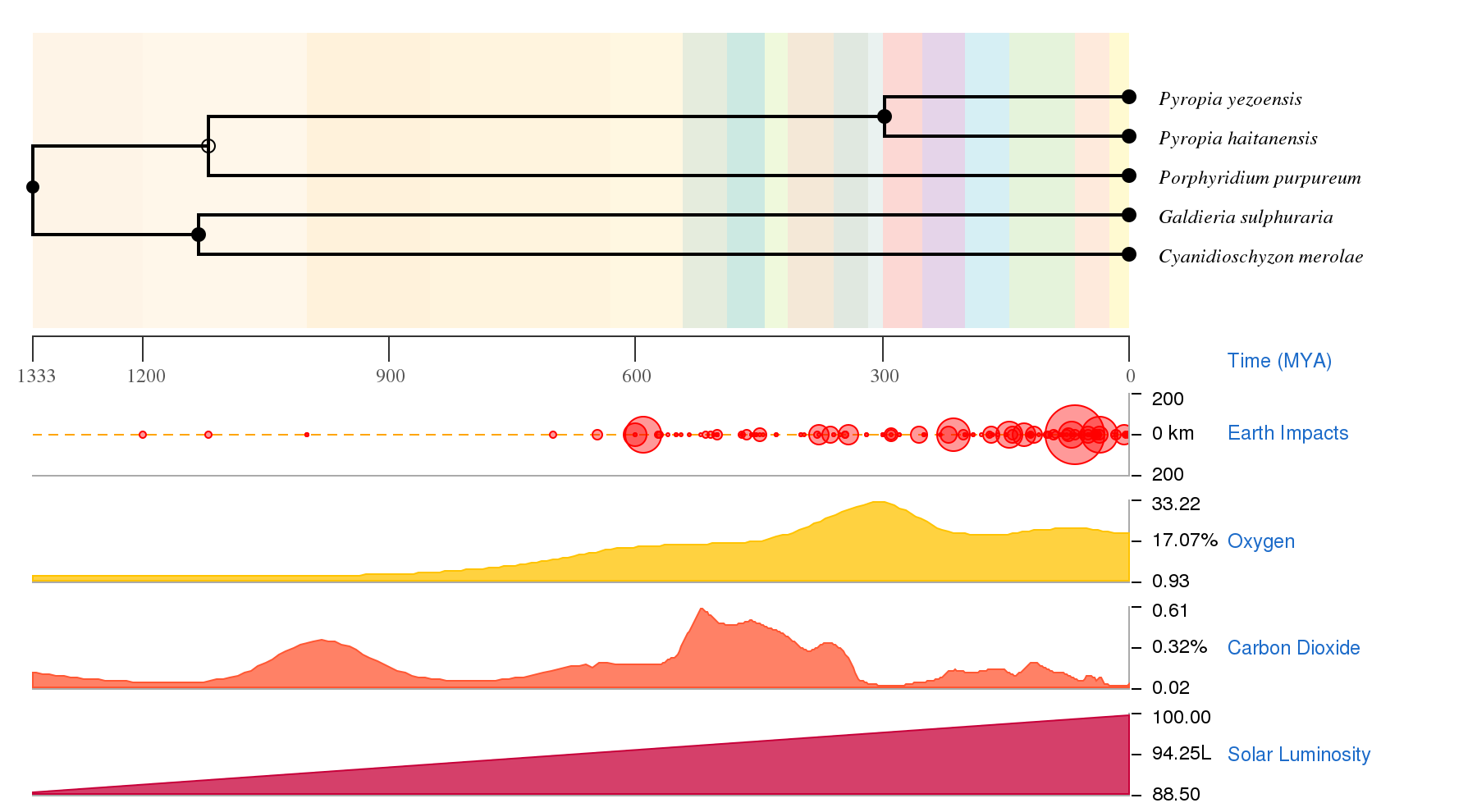

Supplement: FIGURE S4 — The topology of the species evolutionary tree. [file Image_4.TIF]

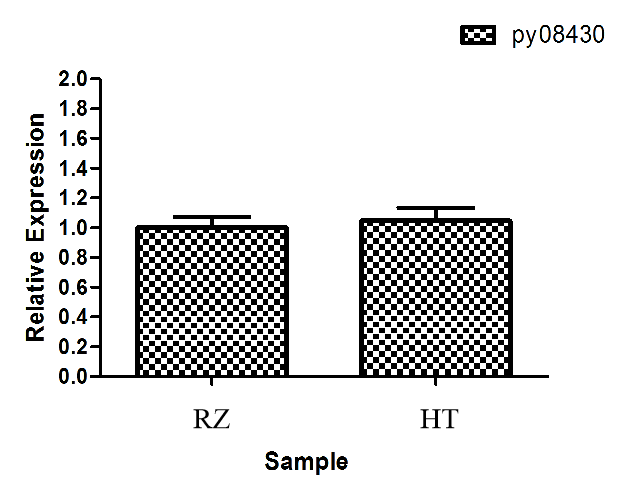

Supplement: FIGURE S5 — Quantitative real-time polymerase chain reaction (qPCR) analysis of Py08430 gene among the wild-type (RZ) and the red mutant (HT). The mean ± SD of the three biological replicates is presented. [file Image_5.PNG]

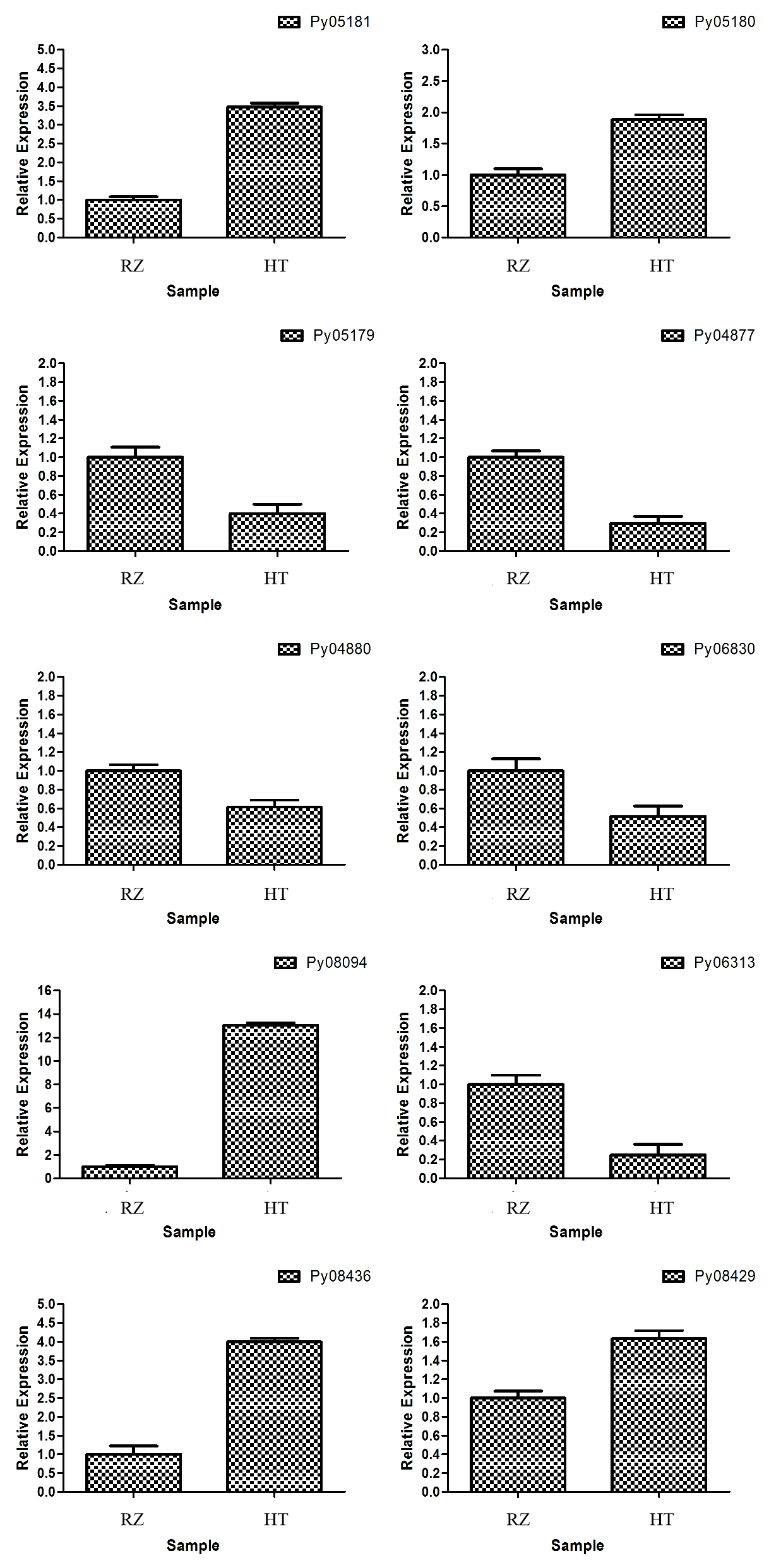

Supplement: FIGURE S6 — Quantitative real-time polymerase chain reaction (qPCR) analysis of 10 genes among the wild-type (RZ) and the red mutant (HT). The mean ± SD of the three biological replicates is presented. [file Image_6.PNG]
