# Supplementary material for: Fine Mapping to Identify the Functional Genetic Locus for Red Coloration in Pyropia yezoensis Thallus
Source: Front Plant Sci. 2020 Jun 23;11:867. doi: 10.3389/fpls.2020.00867 (PMC7324768; doi:10.3389/fpls.2020.00867)
Supplement: TABLE S1 — Color phenotypes and blade types in F1 gametophytic blades from heterozygous conchocelis in the cross between the WT RZ and the red mutant HT of Pyropia yezoensis. W: wild type (WT); R: red type (RT). [file Table_1.DOCX]

| Blade types | | Number |
| --- | --- | --- |
| Unsectored | |  |
| W |  | 856 |
| R |  | 812 |
| Sectored |  |  |
| WR |  | 2287 |
| RW |  | 2256 |
| WRW |  | 461 |
| RWR |  | 313 |
| WRWR |  | 13 |
| RWRW |  | 13 |
| Total |  | 7011 |

1 Color sectors are shown in the order from the base to the apex.
