# Supplementary material for: Fine Mapping to Identify the Functional Genetic Locus for Red Coloration in Pyropia yezoensis Thallus
Source: Front Plant Sci. 2020 Jun 23;11:867. doi: 10.3389/fpls.2020.00867 (PMC7324768; doi:10.3389/fpls.2020.00867)
Supplement: TABLE S2 — The statistics and evaluation of sequencing data generated from paternal parent and the extreme pools in QTL-seq. [file Table_2.DOCX]

| Sample ID | Clean Reads | Clean Base(bp) | GC(%) | SNP Number |
| --- | --- | --- | --- | --- |
| HT | 51,441,406 | 15,432,421,800 | 51 | 41,083 |
| RT24-Pool | 93,448,327 | 28,034,498,100 | 58 | 37,608 |
| WT24-Pool | 84,045,187 | 25,213,556,100 | 43 | 31,510 |
| RT56-Pool | 112,373,023 | 33,711,906,900 | 50 | 36,411 |
| WT56-Pool | 103,512,782 | 31,053,834,600 | 55 | 36,158 |
