# Supplementary material for: Fine Mapping to Identify the Functional Genetic Locus for Red Coloration in Pyropia yezoensis Thallus
Source: Front Plant Sci. 2020 Jun 23;11:867. doi: 10.3389/fpls.2020.00867 (PMC7324768; doi:10.3389/fpls.2020.00867)
Supplement: TABLE S3 — Statistics of linkage map developed in this study. [file Table_3.DOCX]

| Chr. | Map length (cM) | No. of loci | Average marker interval (cM) |
| --- | --- | --- | --- |
| Chr1 | 179.23 | 94 | 1.91 |
| Chr2 | 162.9 | 74 | 2.20 |
| Chr3 | 130.32 | 75 | 1.74 |
| SUM/mean | 472.45 | 243 | 1.94 |
