# Supplementary material for: Fine Mapping to Identify the Functional Genetic Locus for Red Coloration in Pyropia yezoensis Thallus
Source: Front Plant Sci. 2020 Jun 23;11:867. doi: 10.3389/fpls.2020.00867 (PMC7324768; doi:10.3389/fpls.2020.00867)
Supplement: TABLE S4 — Gene primers designed for KASP. [file Table_4.DOCX]

| Maker names | Chr | SNP  position | Primer Allele X (5´-3´)  Primer Allele Y (5´-3´) | Primer Common  (3´-5´) |
| --- | --- | --- | --- | --- |
| SNP1 | chr1 | 41578129 | ACGGTCGGCACACAGTATAGG | GTGAGATATGCGTCTCGGGGCTT |
|  |  |  | GACGGTCGGCACACAGTATAGA |  |
| SNP2 | chr1 | 42833410 | CTTGCACAACAAGTAAGTATGTAGTTGAAA | CAAAGACGAGGAGTCTTTCTGGGTT |
|  |  |  | GCACAACAAGTAAGTATGTAGTTGAAC |  |
| SNP3 | chr1 | 42835120 | AACTTCCGGTTTGGTTCGGAGCA | AGTCCACCGCTTTGAGGAAAGACAT |
|  |  |  | CTTCCGGTTTGGTTCGGAGCC |  |
| SNP4 | chr1 | 42942954 | CATGATCACGACACTATGGTACGTT | GGTTCTCGGAGGCTTGAGAAAAGTA |
|  |  |  | ATGATCACGACACTATGGTACGTC |  |
