# Supplementary material for: Fine Mapping to Identify the Functional Genetic Locus for Red Coloration in Pyropia yezoensis Thallus
Source: Front Plant Sci. 2020 Jun 23;11:867. doi: 10.3389/fpls.2020.00867 (PMC7324768; doi:10.3389/fpls.2020.00867)
Supplement: TABLE S5 — SNPs in candidate region by QTL-seq and KASP. [file Table_5.DOCX]

| Makers | Genome gocation | Related genes | Chr | SNP position | SNP type of RZ | SNP type of HT |
| --- | --- | --- | --- | --- | --- | --- |
| Chr_1_41578129 | intronic | py06969.t1 | 1 | 41578129 | G | A |
| Chr_1_41595872 | intergenic | py06969.t1(dist=17649),py05181.t1(dist=26266) | 1 | 41595872 | G | A |
| Chr_1_41600119 | intergenic | py06969.t1(dist=21896),py05181.t1(dist=22019) | 1 | 41600119 | C | T |
| Chr_1_41772396 | intergenic | py10706.t1(dist=7293),py10705.t1(dist=21287) | 1 | 41772396 | G | T |
| Chr_1_41776954 | intergenic | py10706.t1(dist=11851),py10705.t1(dist=16729) | 1 | 41776954 | C | T |
| Chr_1_41778597 | intergenic | py10706.t1(dist=13494),py10705.t1(dist=15086) | 1 | 41778597 | G | C |
| Chr_1_41848316 | intergenic | py10699.t1(dist=16000),py10698.t1(dist=14934) | 1 | 41848316 | T | G |
| Chr_1_41916015 | intergenic | py10690.t1(dist=16315),py10689.t1(dist=40330) | 1 | 41916015 | G | C |
| Chr_1_41993858 | intergenic | py10688.t1(dist=29784),py02615.t1(dist=79103) | 1 | 41993858 | T | G |
| Chr_1_42298288 | downstream | py04887.t1(dist=427) | 1 | 42298288 | A | G |
| Chr_1_42446782 | intergenic | py04913.t1(dist=3730),py06825.t1(dist=41274) | 1 | 42446782 | C | T |
| Chr_1_42450494 | intergenic | py04913.t1(dist=7442),py06825.t1(dist=37562) | 1 | 42450494 | A | G |
| Chr_1_42476474 | intergenic | py04913.t1(dist=33422),py06825.t1(dist=11582) | 1 | 42476474 | T | C |
| Chr_1_42513943 | intergenic | py06825.t1(dist=25355),py06826.t1(dist=30051) | 1 | 42513943 | T | A |
| Chr_1_42514475 | intergenic | py06825.t1(dist=25887),py06826.t1(dist=29519) | 1 | 42514475 | G | T |
| Chr_1_42515334 | intergenic | py06825.t1(dist=26746),py06826.t1(dist=28660) | 1 | 42515334 | C | G |
| Chr_1_42525501 | intergenic | py06825.t1(dist=36913),py06826.t1(dist=18493) | 1 | 42525501 | C | G |
| Chr_1_42525536 | intergenic | py06825.t1(dist=36948),py06826.t1(dist=18458) | 1 | 42525536 | A | G |
| Chr_1_42528191 | intergenic | py06825.t1(dist=39603),py06826.t1(dist=15803) | 1 | 42528191 | T | C |
| Chr_1_42575018 | intergenic | py06828.t1(dist=19621),py06829.t1(dist=4338) | 1 | 42575018 | T | C |
| Chr_1_42589713 | intergenic | py06830.t1(dist=3912),py06831.t1(dist=3864) | 1 | 42589713 | C | A |
| Chr_1_42645256 | intergenic | py06832.t1(dist=38004),py08099.t1(dist=7869) | 1 | 42645256 | G | A |
| Chr_1_42833410 | upstream | py08429.t1,py08430.t1(dist=719) | 1 | 42833410 | T | G |
| Chr_1_42835120 | exonic | py08429.t1 | 1 | 42835120 | A | C |
| Chr_1_42876706 | intergenic | py08423.t1(dist=15296),py03563.t1(dist=39586) | 1 | 42876706 | A | C |
| Chr_1_42876767 | intergenic | py08423.t1(dist=15357),py03563.t1(dist=39525) | 1 | 42876767 | G | C |
| Chr_1_42942954 | intergenic | py03558.t1(dist=8360),py03556.t1(dist=70285) | 1 | 42942954 | A | G |
| Chr_1_42956204 | intergenic | py03558.t1(dist=21610),py03556.t1(dist=57035) | 1 | 42956204 | C | G |
