# Supplementary material for: Fine Mapping to Identify the Functional Genetic Locus for Red Coloration in Pyropia yezoensis Thallus
Source: Front Plant Sci. 2020 Jun 23;11:867. doi: 10.3389/fpls.2020.00867 (PMC7324768; doi:10.3389/fpls.2020.00867)
Supplement: TABLE S6 — The statistics of sequencing data and genome comparison in RNA-seq. [file Table_6.DOCX]

| Sample | Total Clean Reads (M) | Total Clean Bases(Gb) | Total Mapping(%) |
| --- | --- | --- | --- |
| HT_0 | 37.97 | 11.39 | 90.2% |
| HT_1 | 17.98 | 5.39 | 89.9% |
| HT_3 | 21.50 | 6.45 | 89.9% |
| RZ_0 | 41.13 | 12.339 | 84.5% |
| RZ_1 | 27.32 | 8.19 | 91.3% |
| RZ_2 | 30.91 | 9.27 | 91.0% |
