# Supplementary material for: Fine Mapping to Identify the Functional Genetic Locus for Red Coloration in Pyropia yezoensis Thallus
Source: Front Plant Sci. 2020 Jun 23;11:867. doi: 10.3389/fpls.2020.00867 (PMC7324768; doi:10.3389/fpls.2020.00867)
Supplement: TABLE S8 — Differentially expressed genes in the candidate region of P. yezoensis chromosome 1. [file Table_8.DOCX]

| Genes | Position |
| --- | --- |
| Py05181 | Chr1:41622138-41622968 |
| Py05180 | Chr1:41624845-41626065 |
| Py05179 | Chr1:41627568-41630081 |
| Py04877 | Chr1:42196775-42197575 |
| Py04880 | Chr1:42255454-42255529 |
| Py04907 | Chr1:42420077-42421435 |
| Py06830 | Chr1:42583079-42585801 |
| Py08094 | Chr1:42701806-42703176 |
| Py06313 | Chr1:42766184-42766665 |
| Py08436 | Chr1:42816193-42817467 |
| Py08435 | Chr1:42819126-42820070 |
| Py08433 | Chr1:42822667-42825943 |
| Py08429 | Chr1:42834129-42835466 |
