# Supplementary material for: Fine Mapping to Identify the Functional Genetic Locus for Red Coloration in Pyropia yezoensis Thallus
Source: Front Plant Sci. 2020 Jun 23;11:867. doi: 10.3389/fpls.2020.00867 (PMC7324768; doi:10.3389/fpls.2020.00867)
Supplement: TABLE S9 — Protein databases in NCBI were used for searching homologs of rcl-1. [file Table_9.DOCX]

| Species | Protein databases’ version |
| --- | --- |
| *Arabidopsis thaliana* | TAIR10.1 |
| *Chlamydomonas reinhardtii* | v3.0 |
| *Chlorella variabilis* | v 1.0 |
| *Chondrus crispus* | ASM35022v2 |
| *Cyanidioschyzon merolae strain 10D* | ASM9120v1 |
| *Cyanophora paradoxa* | ASM443141v1 |
| *Ectocarpus siliculosus* | ASM31002v1 |
| *Micromonas pusilla CCMP1545* | v2.0 |
| *Nannochloropsis oceanica* | ASM451948v1 |
| *Oryza sativa* | Build 4.0 |
| *Phaeodactylum tricornutum* | ASM15095v2 |
| *Physcomitrella patens* | Phypa V3 |
| *Porphyra umbilicalis* | P_umbilicalis_v1 |
| *Porphyridium purpureum* | GCA_000397085.1 |
| *Pyropia haitanensis* | OUC_PyHait |
| *Thalassiosira pseudonana* | ASM14940v2 |
| *Volvox carteri* | v1.0 |
| *Zosma marina* | ASM90016980v1 |
