# Supplementary material for: Fine Mapping to Identify the Functional Genetic Locus for Red Coloration in Pyropia yezoensis Thallus
Source: Front Plant Sci. 2020 Jun 23;11:867. doi: 10.3389/fpls.2020.00867 (PMC7324768; doi:10.3389/fpls.2020.00867)
Supplement: TABLE S10 — Primers used in qRT-PCR for validating differentially expressed genes. [file Table_10.DOCX]

| Genes | Prime sequence (forward/reverse, 5'-3') |
| --- | --- |
| *Py04877* | GCACCTTCCTCAACTCCCTC/ AGGCGGTTATCGTCCAATCC |
| *Py04880* | CACCTTCCCACTCTTGCCTT/ GTCCGTGTTGCCCTTCTCC |
| *Py05179* | TGGCGGCGATGGGTATGA/ GTGGAGGCAGTGGCAGTAG |
| *Py05181* | CCCTCCGCTTGCTTCTATCC/ GCCTCGTCCGCTTTCTTTGA |
| *Py05180* | AGGCACGGGAGCAGGATACA/ CGCAGACCTCGCACATAAAC |
| *Py06313* | CCGCTCGGAACTACCCTG/ CCTCGCTCTTCTCCACCTTG |
| *Py06830* | GAGGCGATGGAGCAGCAGA/ GTCAAAGCGGGAGAGGATGG |
| *Py08094* | AGGATGGCAGTTCAAGCGA/ GAGCAGAGATGACAAAAGCGG |
| *Py08436* | CGCCGAGCATTCCAAGT/ GTCACCCGCACAGCAGA |
| *Py08429* | GCCATTGCGTGCTACCTTGC/ CCTCGGGAGCCACCGAGA |
| *UBC* | TCACAACGAGGATTTACCACC/ GAGGAGCACCTTGGAAACG |
| *Py08430* | ACCGTATCACTATCCCCCGA/ CGCTCTCGGTAGGCAGT |
